# Supplementary material for: GSDME-mediated pyroptosis promotes the progression and associated inflammation of atherosclerosis
Source: Nat Commun. 2023 Feb 18;14:929. doi: 10.1038/s41467-023-36614-w (PMC9938904; doi:10.1038/s41467-023-36614-w)
Supplement: Supplementary file 1 — Supplementary Information [file 41467_2023_36614_MOESM1_ESM.pdf]

Supplementary Information

GSDME-mediated Pyroptosis Promotes the Progression and Proinflammation of Atherosclerosis

Yuanyuan Wei[1], Beidi Lan[1,2], Tao Zheng [1,2], Lin Yang[3], Xiaoxia Zhang[4], Lele Cheng[1, 2], Gulinigaer Tuerhongjiang[1,2], Zuyi Yuan[1,2*], Yue Wu[1,2*]

1. Department of Cardiovascular Medicine, The First Affiliated Hospital of Xi'an Jiaotong University, Xi’an, Shaanxi, China;
2. Key Laboratory of Environment and Genes Related to Diseases, Ministry of Education, Xi'an Jiaotong University, Xi’an, Shaanxi, China;
3. Department of Vascular Surgery, The First Affiliated Hospital, Xi'an Jiaotong University, Xi'an, Shaanxi, China;
4. Department of Pharmacy, The First Affiliated Hospital, Xi'an Jiaotong University, Xi'an, Shaanxi, China.

Contents:

Supplementary Figures

Reagents used in the study

Supplementary Table 1 - Clinical characteristics of the patients

Supplementary Table 2 - Specific primers for quantitative RT-PCR

Supplementary Table 3 - Primers for chip-PCR

List of supplementary figures

Supplementary Fig.1 Pathology of atherosclerotic plaques.

Supplementary Fig.2 Single-cell transcriptome analysis of human advanced carotid artery atherosclerotic plaques. Cells with over 10% mitochondrial gene content were removed.

Supplementary Fig. 3 Gene ontology (GO) analysis of genes differentially expressed in M1 or M2 macrophages.

Supplementary Fig. 4 Subtypes among myeloid cells, especially M1 and M2 macrophages demonstrate important differences in human atherosclerosis.

Supplementary Fig. 5 *GSDME* is mainly expressed in atherosclerotic macrophages. Cells with over 50% mitochondrial gene content were removed.

Supplementary Fig. 6 Single-cell transcriptome analysis of human advanced carotid artery atherosclerotic plaques. Cells with over 50% mitochondrial gene content were removed.

Supplementary Fig. 7 The protein expression of GSDME in different parts of the same plaque.

Supplementary Fig. 8 Analysis of the content of collagen, macrophages, and smooth muscle cells in atherosclerotic plaques.

Supplementary Fig. 9 Serum levels of interleukin (IL)-1β, IL-6, tumor necrosis factor-α (TNF), and monocyte chemoattractant protein-1 (MCP-1) assayed by Luminex technology (n=8).

Supplementary Fig. 10 Representative hematoxylin-eosin (H&E) and Oil Red O staining image of lesions in sequential aortic sinus sections.

Supplementary Fig. 11 Ox-LDL upregulated GSDME expression in macrophages.

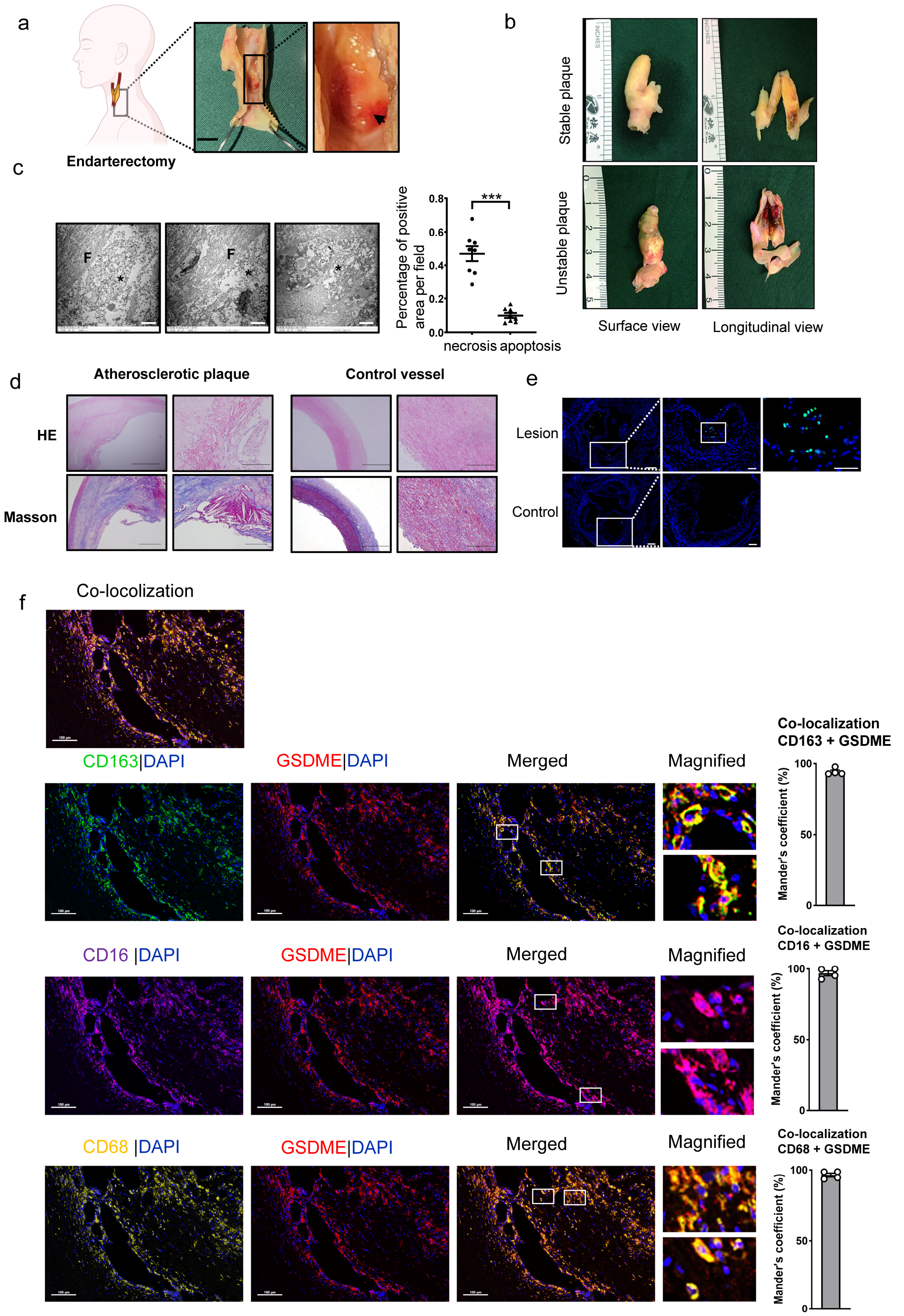

Supplementary Fig. 1 Pathology of human and mouse atherosclerotic plaques. (a) Freshly obtained human plaque samples from endarterectomy procedures. The sample was opened longitudinally for

enface intima visualization. The arrow shows plaque hemorrhage. Scale bar:0.5cm. **(b)** Morphology of stable and unstable carotid atherosclerotic plaques. **(c)** Electronic microscopic appearance of cell death in human carotid atherosclerotic plaques and quantitative electron microscopic evaluation of carotid plaques. Asterisks indicate debris of cell lysis. F marks collagen fibrils in the extracellular matrix. Scale bar: 5μm. Data are expressed as mean± SEM. n=8/group, ***$P < 0.001$ by two-tailed Student's t-test. **(d)** Representative H&E (hematoxylin and eosin) and masson trichrome staining of the carotid atherosclerotic plaques of patients undergoing carotid endarterectomy (n=6/6, plaques/control vessels from one experiment). Scale bar 1mm (left) 500μm (right). **(e)** Representative images of TUNEL (terminal deoxynucleotidyl transferase dUTP nick end labeling) staining of lesions in aortic sinus sections of male $ApoE^{-/-}$ mice fed by a western diet for 12wk (n=3/3, lesion/control from one experiment). Normal cell nuclei are blue; Pyroptotic cell nuclei are green. Arrows point to TUNEL-positive nuclei in atherosclerotic lesions. Scale bar 1mm. **(f)** mIF images of human carotid artery atheroma sections labeled with DAPI (blue), GSDME (red), CD163 (green), CD16 (purple), and CD68 (yellow). n=4, Scale bar: 100 μm. mIF, multiplex immunofluorescence. Data are expressed as mean±SEM. Source data are provided as a Source Data file.

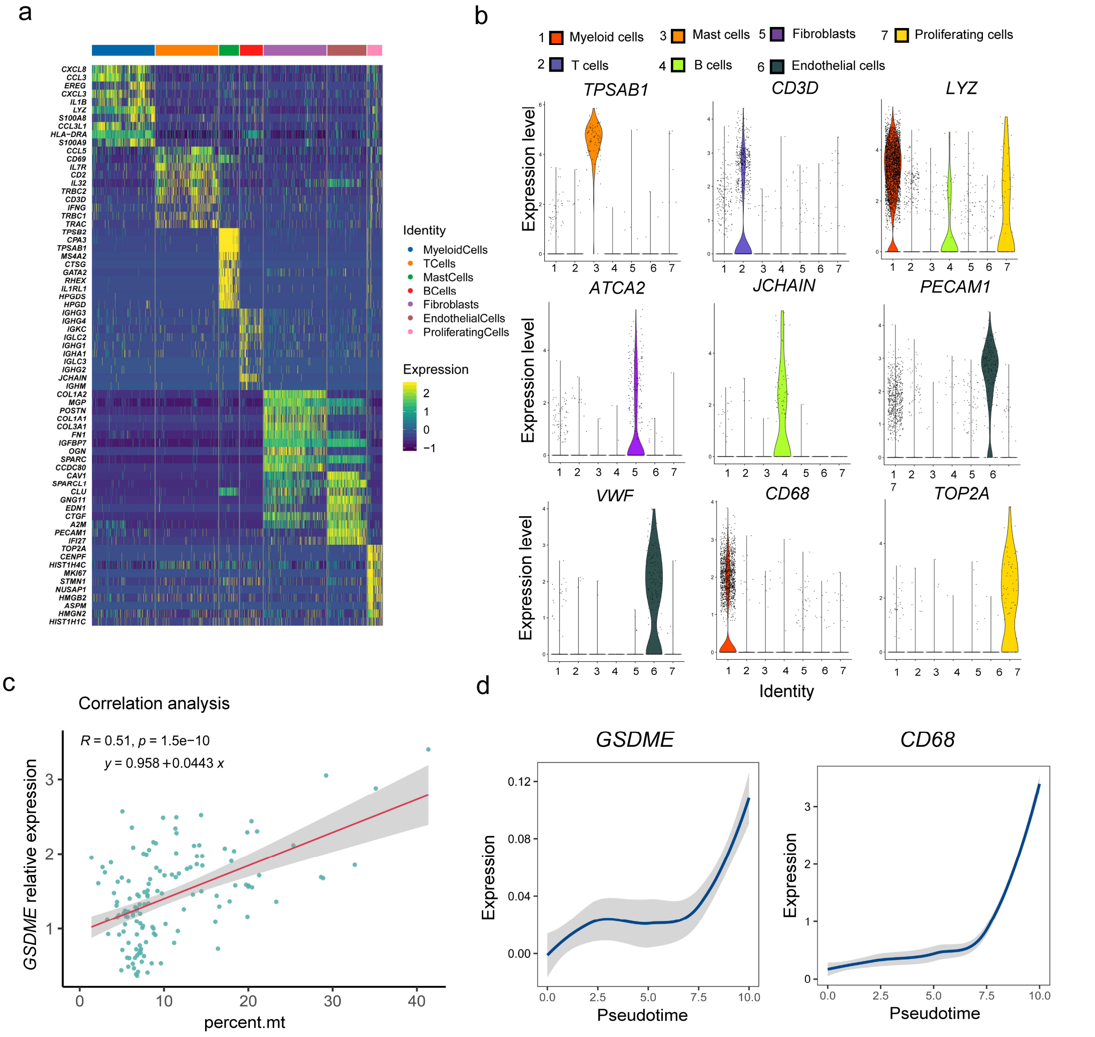

Supplementary Fig. 2 Single-cell transcriptome analysis of human advanced carotid artery atherosclerotic plaques. Cells with over 10% mitochondrial gene content were removed. **(a)** Heatmap of top 10 marker genes per cluster. The color scale represents expression levels; blue: low, yellow: high. **(b)** Violin plots of signature genes confirmed population identities. Cell types are labeled by different colors. **(c)** Correlation analysis between *GSDME* expression and mitochondrial gene content. The red line represents the linear regression mean and the shaded areas indicate 95% confidence intervals. **(d)** Pseudotime developmental analysis of *GSDME* and macrophage marker *CD68* showed the changes during the progression. The fitting curve analysis was performed using the ggplot2 geom_smooth

function default parameters, where the shaded areas are the amount of smoothness in the 95% confidence interval.

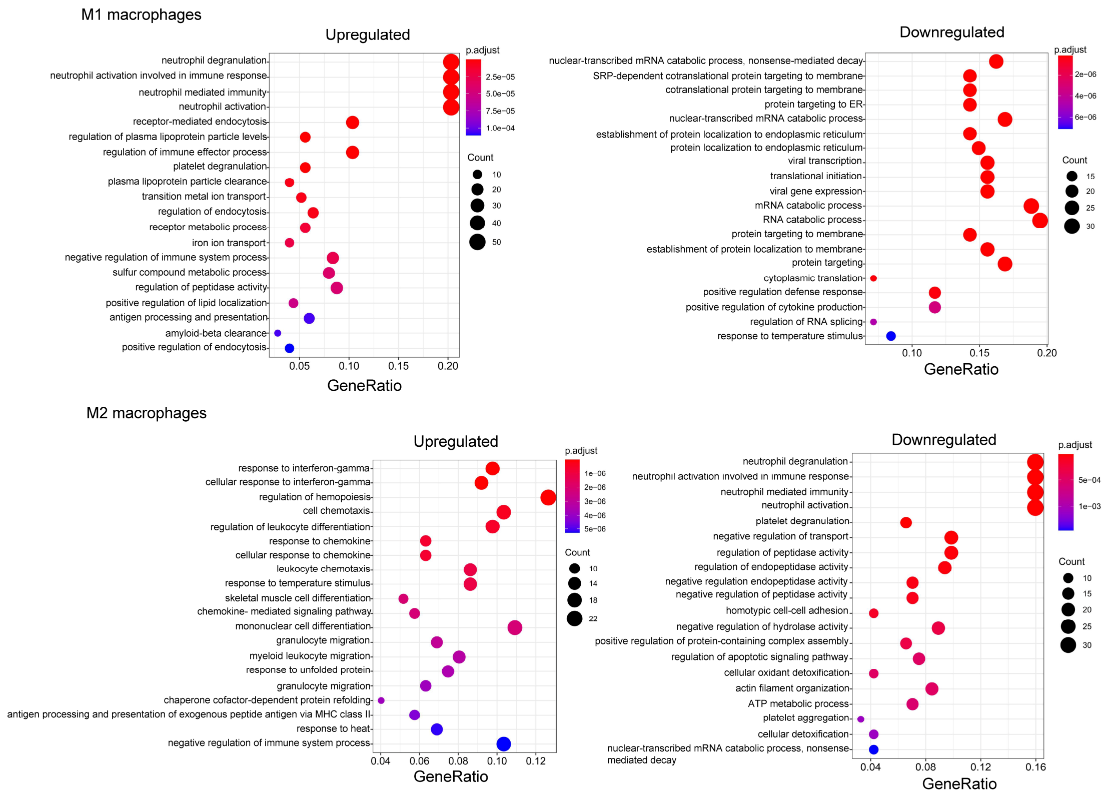

Supplementary Fig. 3 Gene ontology (GO) analysis of genes differentially expressed in M1 or M2 macrophages. Cells with over 10% mitochondrial gene content were removed. Dot plots of significant GO terms are overrepresented by genes with enhanced expression or declined expression in M1 macrophages (Upper) as well as in M2 macrophages (Lower). The color scales represent the adjusted *P* value.

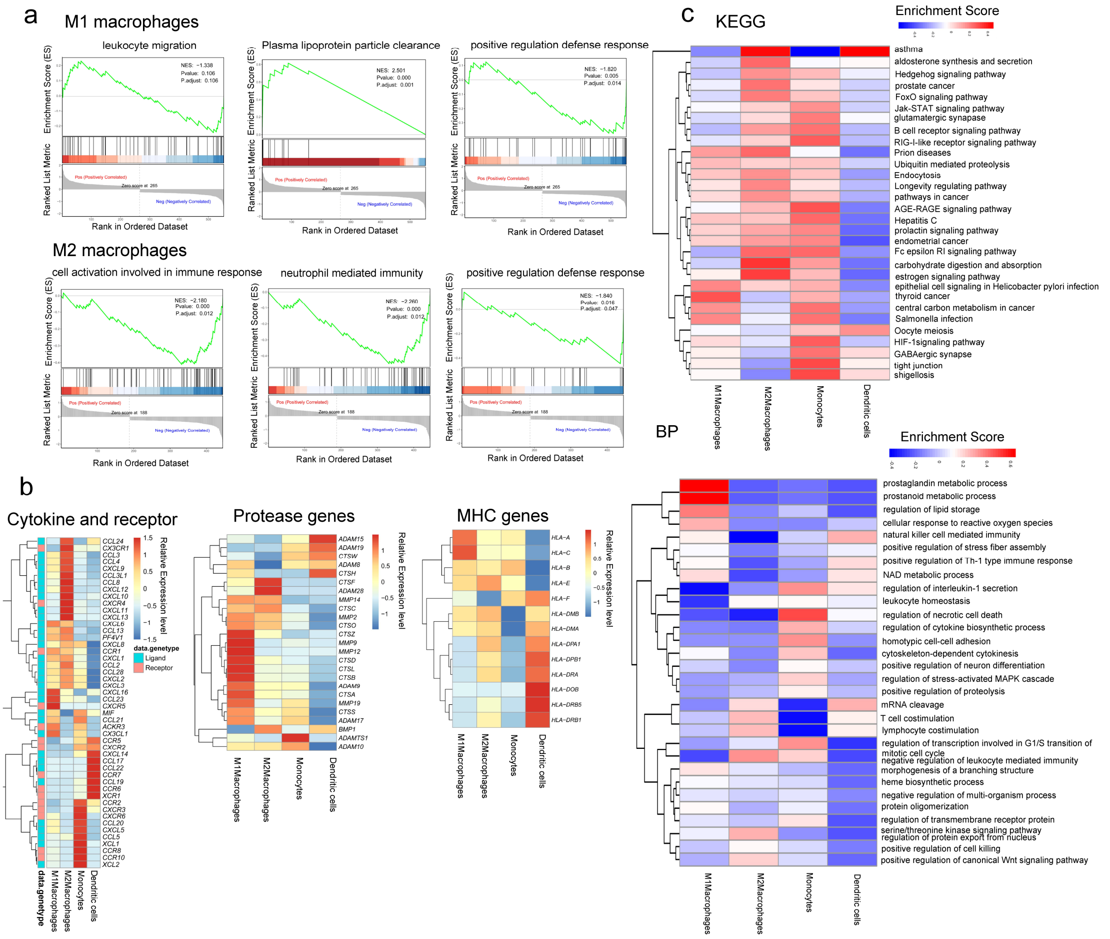

Supplementary Fig. 4 Subtypes among myeloid cells, especially M1 and M2 macrophages demonstrate important differences in human atherosclerosis. Cells with over 10% mitochondrial gene content were removed. **(a)** Gene set enrichment analysis (GSEA) result for the gene set of specifical biological process versus differently expressed genes of M1 macrophage (Upper) and M2 macrophages (Lower). **(b)** Mean expression of cytokine, proteinase, and major histocompatibility complex (MHC) genes across myeloid cell clusters. The color scale represents relative expression levels; blue: low, red: high. **(c)** Gene set variation analysis (GSVA) derived heatmap of differently expressed pathways and biological processes in myeloid cells. The color scale represents the enrichment score; blue: low, red: high. KEGG, Kyoto Encyclopedia of Genes and Genomes. BP, biological process.

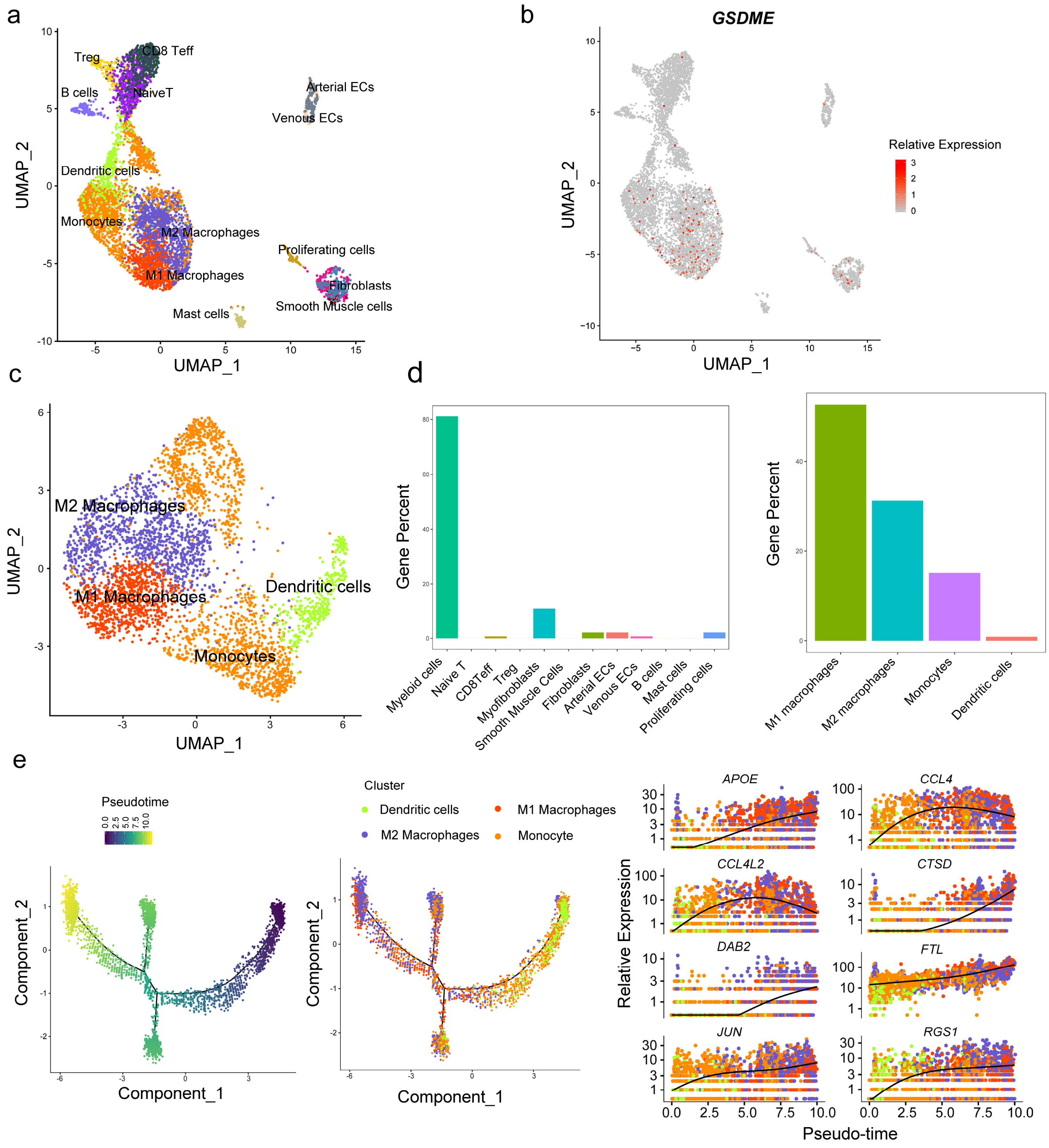

Supplementary Fig. 5 *GSDME* is mainly expressed in atherosclerotic macrophages. Single-cell transcriptomic profiling and dissection of the cellular heterogeneity from human carotid artery advanced atherosclerotic lesions. Cells with over 50% mitochondrial gene content were removed. 6 758 cells from the human carotid artery advanced atherosclerotic lesions of patients undergoing carotid endarterectomy. **(a)** Uniform manifold approximation and projection (UMAP) dimensional reduction of the 6 758 atheroma cells.15 cell types were identified. **(b)** Biaxial scatter plots show the expression pattern of *GSDME* among the different subgroups in the total atheroma cells. Color scale represents relative expression levels; gray: low, red: high. **(c)** UMAP distribution of clustering revealed 4 distinct myeloid

populations. Population identities were determined based on marker gene expression. **(d)** Bar graphs show the expression pattern of *GSDME* among the different subgroups in the total atheroma cells. **(e)** The myeloid cell development trajectory visualization in a biaxial scatter plot. The color scale represents the development stage, and dark colors indicate early development (Left). Pseudotime developmental analysis revealed a branched single-cell trajectory of myeloid cells beginning with monocytes and dendritic cells (Middle). Scatter plots for example differently expressed genes (DEGs) of myeloid cells depicting expression level as a function of pseudo time score. Each point represents a single cell. The color scheme depicts a cluster (Right).

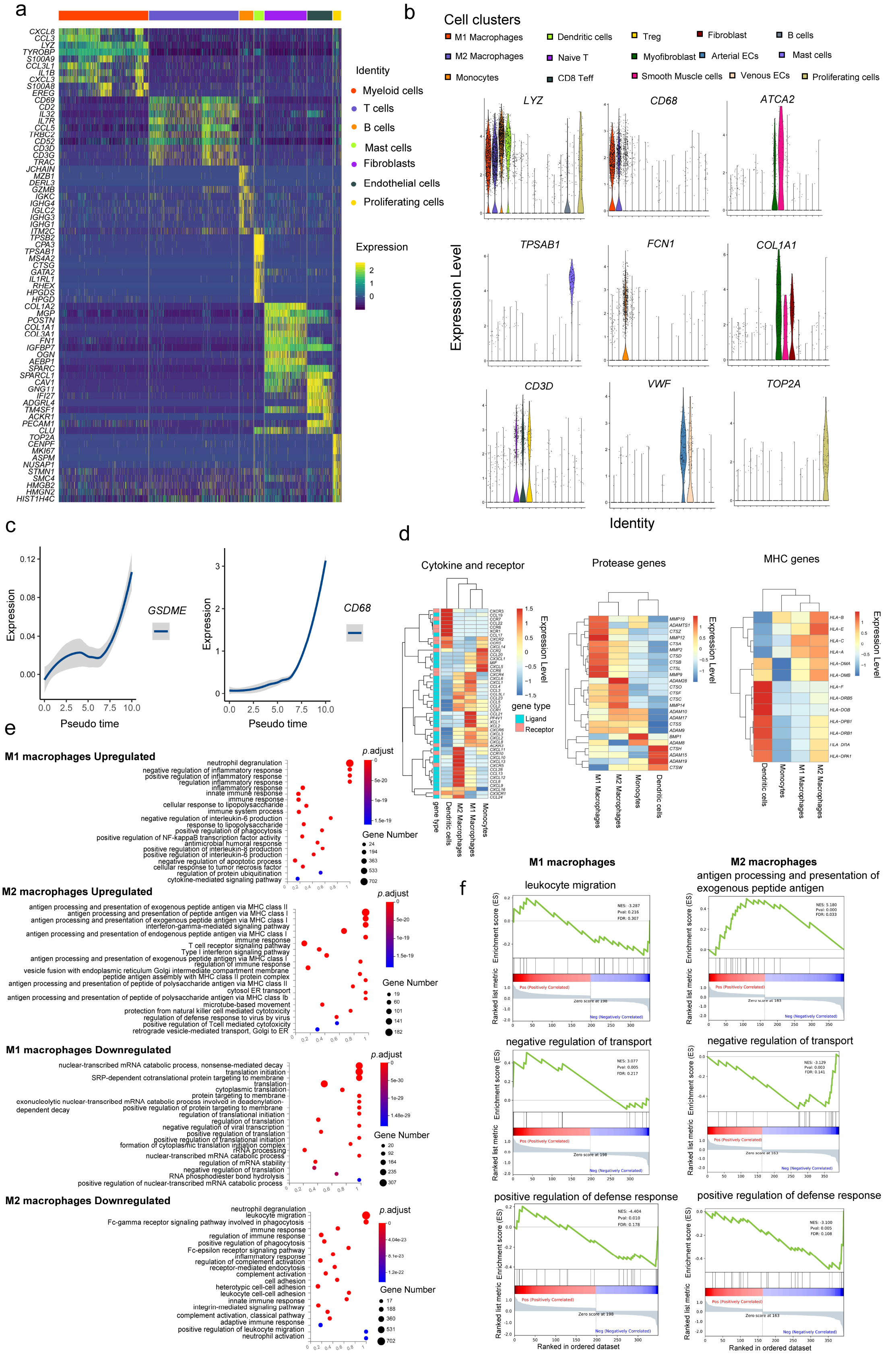

Supplementary Fig. 6 Single-cell transcriptome analysis of human advanced carotid artery atherosclerotic plaques. Cells with over 50% mitochondrial gene content were removed. **(a)** Heatmap

of top 10 marker genes per cluster. **(b)** Violin plots of signature genes confirmed population identities and the *GSDME* gene. **(c)** Pseudotime developmental analysis of *GSDME* and macrophage marker *CD68* showed the changes during the progression. The red line represents the linear regression mean and the shaded areas indicate 95% confidence intervals. **(d)** Mean expression of cytokine, proteinase, and MHC genes across myeloid cell clusters. M1 macrophage expressed several cytokine genes, including *MIF*, *CXCL5*, *CCL21*, and *CCL3*, indicating an inflammatory function. The monocyte cluster highly expressed cytokine receptor genes, including *CCR5*, *CCR2*, and *CCR8*. Similarly, the dendritic cells expressed several receptor genes, including *CXCR3*, *CCR7*, *CCR6*, and *CCR5*. Expression values were row scaled and are shown by color. **(e)** Dot plots of significant GO terms are overrepresented by genes with enhanced expression or declined expression in M1 macrophages as well as in M2 macrophages. Neutrophil activation, leukocyte migration, and inflammatory response were upregulated in M1 macrophages; antigen processing and presentation of peptide antigen, and negative regulation of the immune system were activated in M2 macrophages. Conversely, neutrophil activation, leukocyte migration, and cell chemotaxis were downregulated in M2 macrophages. The color scales represent the adjusted *P* value. **(f)** GSEA result for the gene set of specifical biological process versus differently expressed genes of M1 macrophage (Left) and M2 macrophages (Right). Genes related to leukocyte migration were upregulated in M1 while functions of M2 macrophages leaned towards antigen processing.

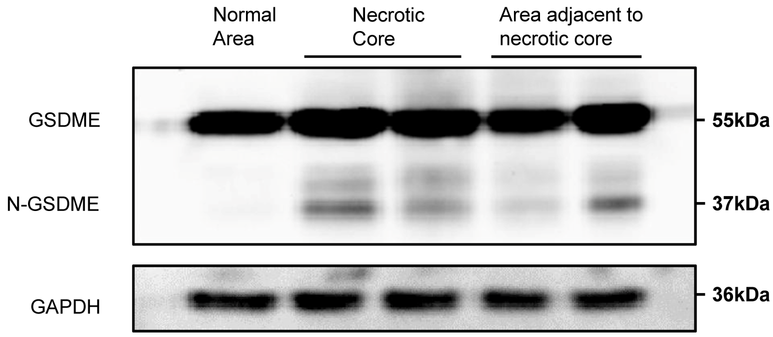

Supplementary Fig. 7 The protein expression of GSDME in different parts of the same plaque.

GSDME and N-GSDME proteins were determined by Western blot in different parts of human carotid artery plaque. The experiment was repeated independently from 3 plaques with similar results. Source data are provided as a Source Data file.

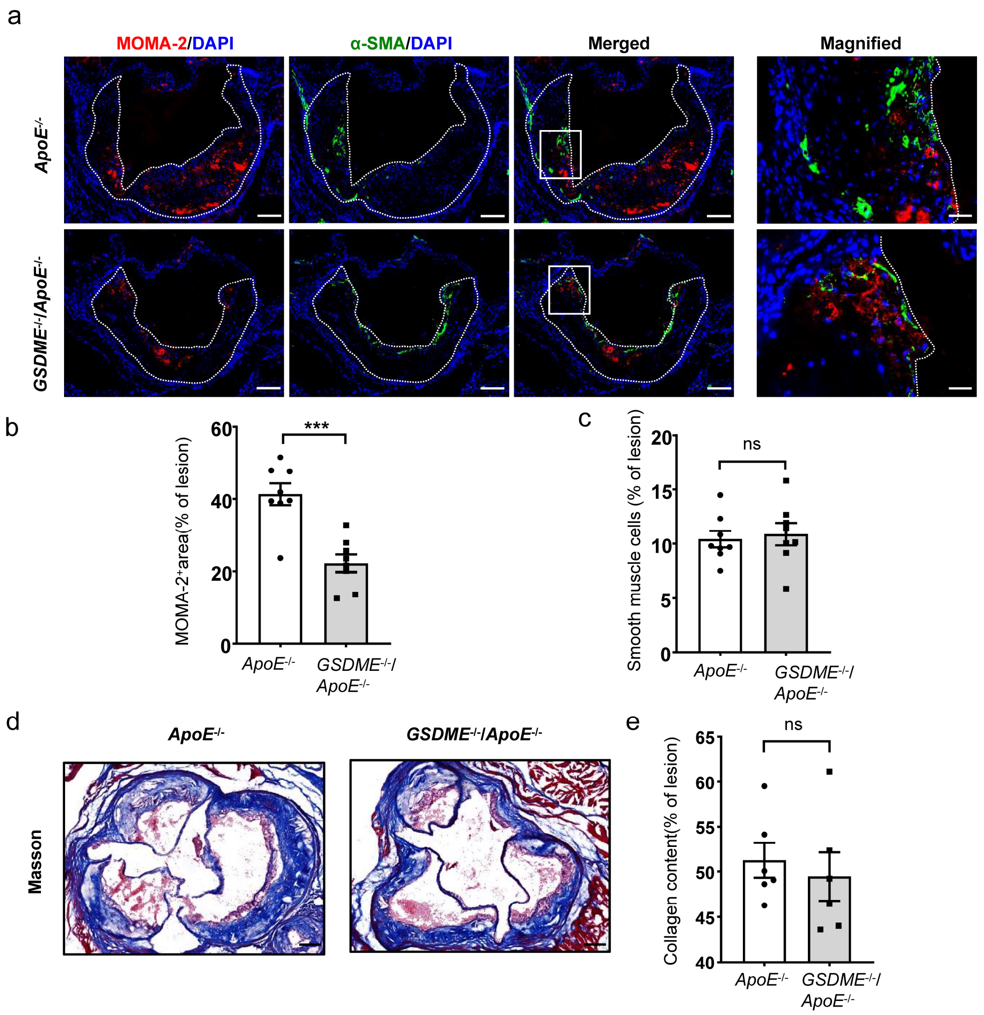

Supplementary Fig. 8 Analysis of the content of collagen, macrophages, and smooth muscle cells in atherosclerotic plaques. Male 8-wk-old *ApoE*$^{-/-}$ and *GSDME*$^{-/-}$/*ApoE*$^{-/-}$ mice were fed a high-fat diet for 12 wk. **(a)** Representative immunofluorescence images of aortic sinus co-stained with α-SMA and MOMA2 antibody. Scale bar: 100 µm; Scale bar: 20 µm（magnification）**(b-c)** Quantification of macrophages and smooth muscle cell content based on MOMA2 (n=8/group, ***$P < 0.001$) and α-SMA (n=8/group, P=0.720)-positive areas in plaques. **(d)** Representative images of Masson trichrome staining for collagen content in the aortic sinus. Scale bar: 200 µm. **(e)** Quantification of collagen content in plaques (n=6/group, P=0.888). ns, not significant. For all panels, data are expressed as mean±SEM.

Unpaired two-tailed Student's *t*-test was used for between-group comparisons. Source data are provided as a Source Data file.

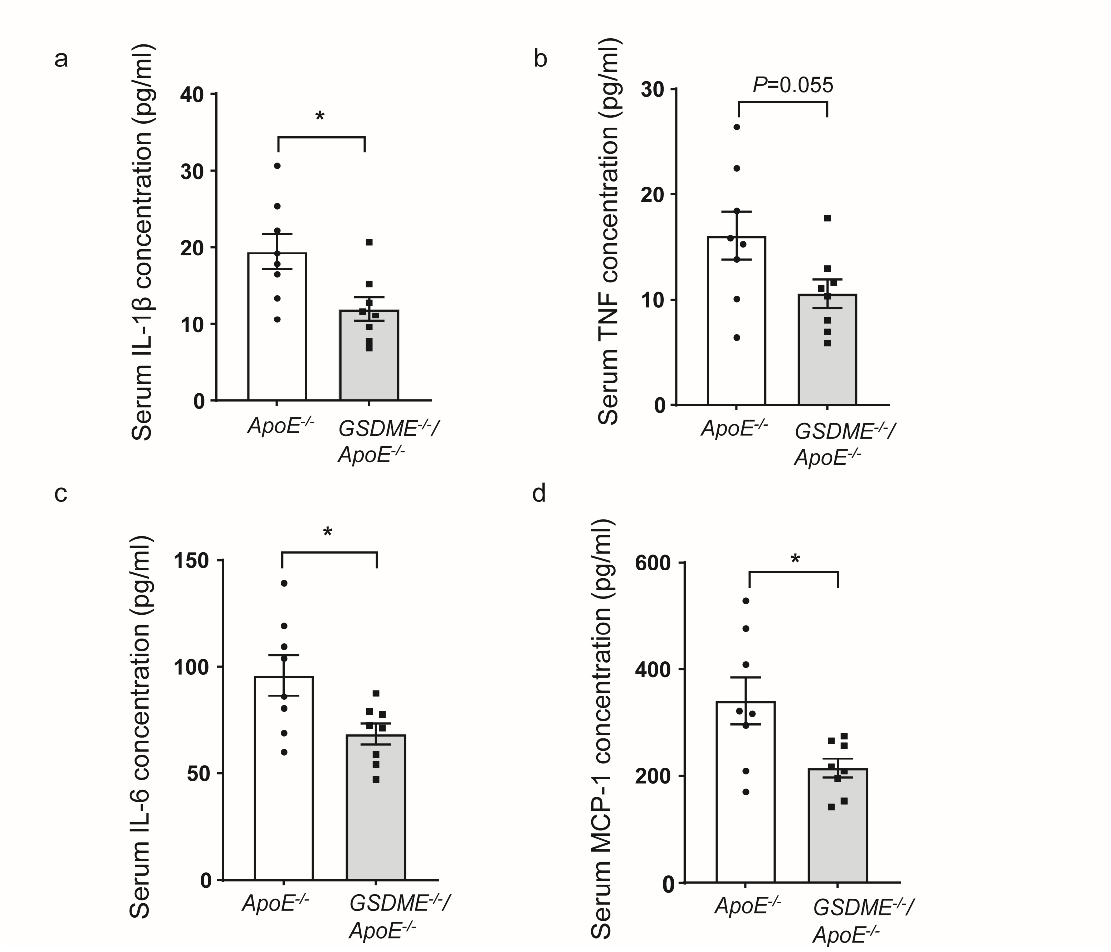

Supplementary Fig. 9 Serum levels of **(a)** Interleukin (IL)-1β, **(b)** Tumor necrosis factor-α (TNF), **(c)** IL-6, and **(d)** Monocyte chemoattractant protein-1 (MCP-1) were assayed by Luminex (n=8/group). IL-1β (**P*=0.017), TNF (*P*=0.055), IL-6 (**P*=0.022), MCP-1 (**P*=0.018). For all panels, data are expressed as mean±SEM. Unpaired two-tailed Student's *t*-test was used for between-group comparisons. Source data are provided as a Source Data file.

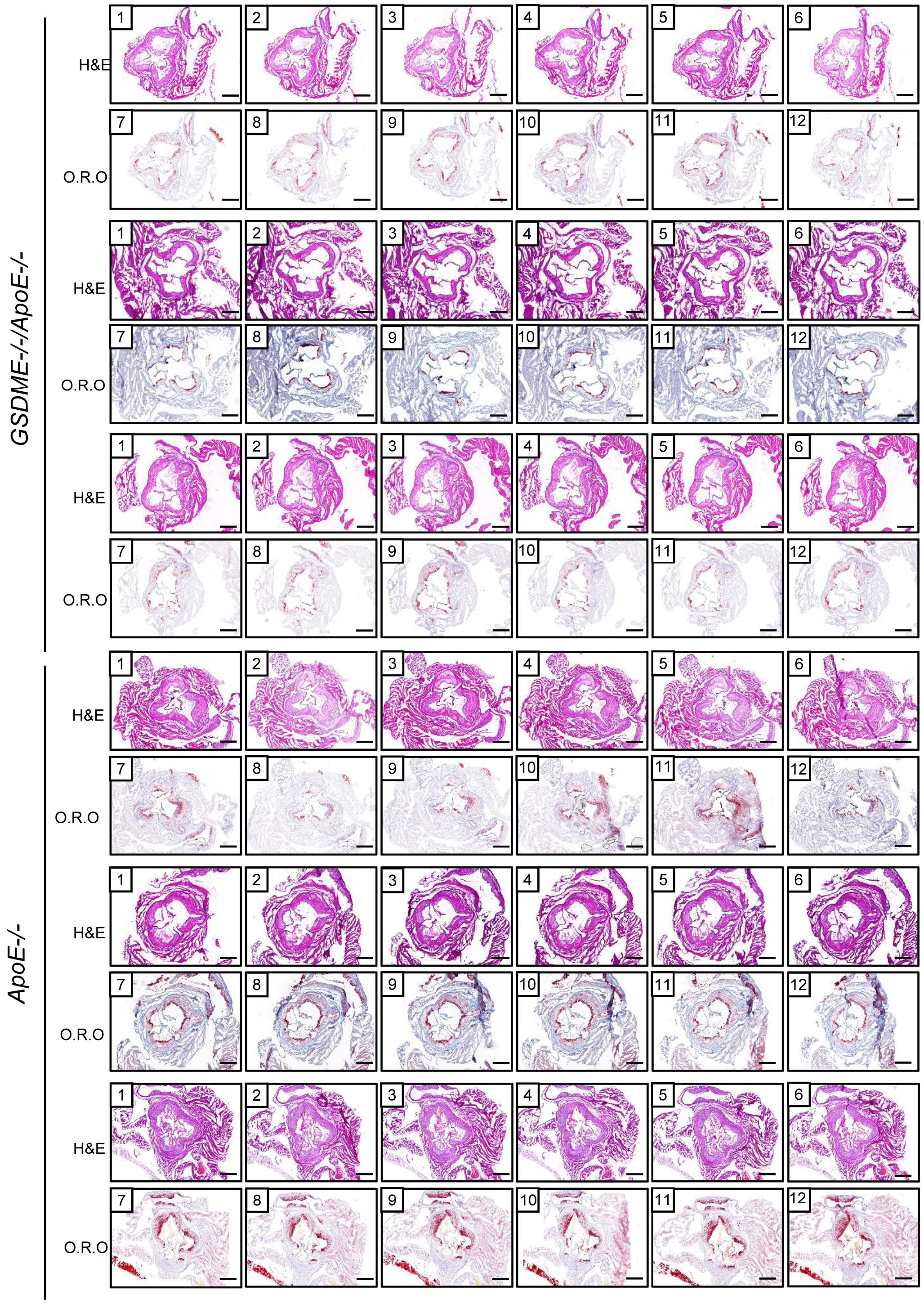

Supplementary Fig. 10 Representative hematoxylin-eosin (H&E) and Oil Red O staining image of lesions in sequential aortic sinus sections from *GSDME*$^{-/-}$/*ApoE*$^{-/-}$ or *ApoE*$^{-/-}$ mice (n=10/8, *ApoE*$^{-/-}$/ *GSDME*$^{-/-}$/*ApoE*$^{-/-}$ mice from one experiment. Scale bar: 500μm.

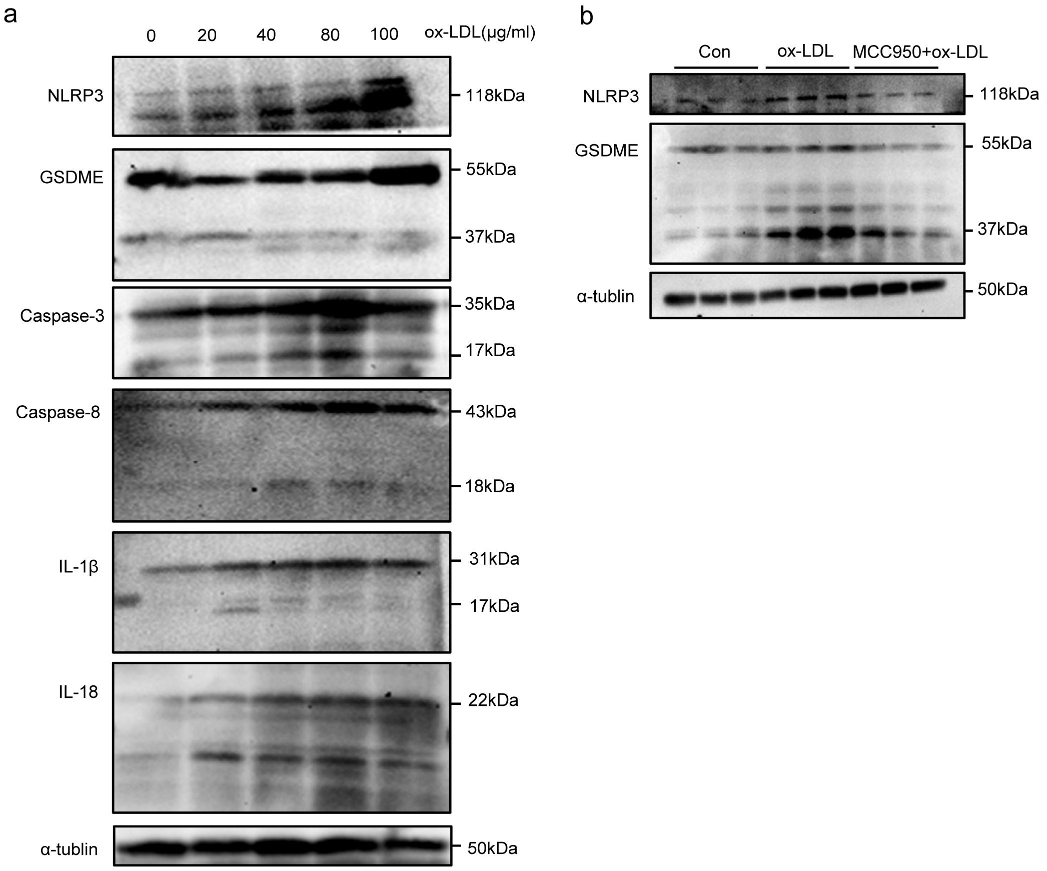

Supplementary Fig. 11 ox-LDL upregulated GSDME expression in macrophages. **(a)** ox-LDL (0, 20 40, 80, or 100 μg/ml) were incubated with peritoneal macrophages (PMs) for 24h. The expression of NLRP3, GSDME, caspase-3, caspase-8, IL-18, and IL-1β were measured by western blotting. **(b)** The efficiency of MCC950, and its effect on GSDME activation and expression. Cells were pretreated with MCC950 (10 μmol/L) for 4 hours and then exposed to ox-LDL (100 μg/ml) for a further 24 hours. Each experiment was repeated independently 3 times for **(a-b).** Source data are provided as a Source Data file

Reagent used in the study

Anti-GSDME (Abcam, catalog number: ab230482, 1:100, Rabbit polyclonal), anti-caspase-3 (CST, catalog number: 9662, 1:1000, Rabbit polyclonal), anti-IL-1β (Proteintech, catalog number: 66737-1-Ig, 1:200, Clone numbers: 2A1B4), anti-CD68 (Proteintech, catalog number: 66231-2-Ig, 1:2000, Clone numbers: 3A9A7), anti-Anti-Monocyte + Macrophage (Abcam, catalog number: ab33451, 1:1000, Clone numbers: MOMA-2), α-SMA (CST, catalog number: 19245, 1:200, Clone numbers: D4K9N), anti-CD16 (Abcam, catalog number 183354, 1:100, Clone numbers: SP175) and anti-CD163 (Abcam, catalog number 182422, 1:200, Clone numbers: EPR19518) were used for immunofluorescence and immunohistochemistry staining. Anti-GSDME (Abcam, catalog number: ab215191, 1:1000, Clone numbers: EPR19859), anti-caspase-3 (CST, catalog number: 9662, ab230482, 1:1000, Rabbit polyclonal), anti-α-tubulin (Affinity Biosciences, catalog number: AF7010, Rabbit polyclonal), anti -STAT3 (CST, catalog number: 12640, 1:1000, Clone numbers: D3Z2G), anti-phospho-STAT3 (CST, catalog number:9145, 1:1000, Clone numbers: D3A7), anti-IL-1β (CST, catalog number: 12242, 1:1000, Clone numbers: 3A6), normal Rabbit IgG (CST, catalog number:2729, 5 μg for a single immunoprecipitation assay), anti-FLAG (CST, catalog number: 14793, 1:1000, Clone numbers: D6W5B), anti-GAPDH (Affinity Biosciences, catalog number: AF7021, Rabbit polyclonal), anti-NLRP3 (Abcam, catalog number: ab270449, 1:1000, Clone numbers: EPR23073-96), anti-caspase-8 (CST, catalog number:8592 1:1000, Clone numbers: D5B2), anti-rabbit IgG, HRP-linked Antibody (CST, catalog number:7074, 1:2000) were used for Western blotting. Cycloheximide (catalog number: 14126) was from Cayman Chemical (Ann Arbor, MI). MCC950 (catalog number: HY-12815) was from MCE (USA). Recombinant mouse TNF (catalog number: AF-315-01A) was from PeproTech (USA). Human ox-LDL(catalog number: YB-002) was purchased from Yiyuan Biotech (Guangzhou, China). MiniBEST Universal RNA

extraction kit (catalog number: 9767), and PrimeScript RT Master Mix (catalog number: RR036A) were purchased from Takara Bio (Japan). FastStart Essential DNA Green (catalog number: 06402712001) was purchased from Roche Diagnostics (Mannheim, Germany). Duolink In situ Brightfield kit (catalog number: DUO 92012) was purchased from Sigma-Aldrich (St. Louis, MO). The Cytotoxic Non-Radioactive Cytotoxicity Assay kit (catalog number: G1780) and Dual-luciferase Reporter Assay kit (catalog number: E1910) were purchased from Promega (Madison, USA). SimpleChIP Enzymatic Chromatin IP Kit（catalog number: #9003）was purchased from Cell Signaling Technology (CST, Danvers, MA).

Supplementary Table 1. Clinical characteristics of the patients used in Fig. 2 and Fig. 3.

	**Patient1**	**Patient2**	**Patient3**	**Patient4**	**Patient5**
Sex	M	M	M	M	M
Age	63	60	71	67	75
BMI	23.87	25.83	24.67	25.36	25.95
Smoking	Past (quit for 20 years)	Past (quit for 3 years)	Never	Past (quit for 10 years)	Never
Hypertension	Y	Y	F	F	Y
Diabetes	Y	Y	F	F	F
Surgery	Right carotid CEA	Right carotid CEA	Left carotid CEA	Left carotid CEA	Left carotid CEA
Main complaints	Physical examination reveals carotid stenosis	Physical examination reveals carotid stenosis	Alalia; Weakness of right upper limb	Dizziness; Weakness of right upper limb	Anandia; Cognitive dysfunction
Indications for atherectomy	Stroke; Lumen stenosis: 50-60%	Stroke; Lumen stenosis: 70-80%	Stroke; Lumen stenosis:70-90%	Stroke; Lumen stenosis: 70-90%	Stroke; Lumen stenosis:70-90%
BG (mmol/L)	7.50	5.45	6.06	4.44	3.07
SBP/DBP (mmHg)	141/79	211/82	130/80	145/87	137/76
HDL (mmol/L)	0.67	0.89	0.53	0.94	0.97
LDL (mmol/L)	0.93	2.73	1.57	2.22	1.74
TG (mmol/L)	0.68	1.43	1.94	2.17	1.75
TC (mmol/L)	2.04	4.08	2.75	3.96	3.33

	Patient6	Patient7	Patient8	Patient9*	Patient10*
Sex	M	F	M	M	F
Age	55	50	55	60	41
BMI	26.37	22.86	27.68	24.49	23.44
Smoking	Current	Never	Current	Current	Never
Hypertension	Y	F	Y	F	F
Diabetes	Y	F	F	F	F
Surgery	Right carotid CEA	Left carotid CEA	Right carotid CEA	Left carotid CEA	Left carotid CEA
Main complaints	Dizziness	Dizziness; Cephalalgia	Numbness and weakness of left upper limb	Dizziness	Dizziness
Indications for atherectomy	TIA; Lumen stenosis: 90%	TIA; Lumen stenosis: 50-60%	Stroke; Lumen stenosis: 70-90%	Stroke; Lumen stenosis:50-70%	Stroke; Lumen stenosis: 90%
BG (mmol/L)	5.35	4.12	4.04	11.75	3.93
SBP/DBP (mmHg)	119/90	120/71	127/80	139/87	130/70
HDL (mmol/L)	0.83	0.95	0.97	0.75	1.56
LDL (mmol/L)	2.78	1.55	2.93	3.61	2.80
TG (mmol/L)	1.82	0.76	2.76	1.03	2.06
TC (mmol/L)	4.20	3.29	4.73	4.68	5.04

Atherosclerotic plaques were obtained from 8 male and 2 female patients undergoing a carotid endarterectomy (CEA) procedure in The First Affiliated Hospital of Xi'an Jiao tong University; BG: blood glucose; SBP: systolic blood pressure; DBP: diastolic blood pressure; HDL cholesterol: high-density lipoprotein cholesterol; LDL cholesterol: low-density lipoprotein cholesterol; TG: triglyceride; TC: total cholesterol; TIA: Transient Ischemic Attack; *Patients' information used in single-cell analysis.

Supplementary Table 2. Specific primers for quantitative RT-PCR

Gene name	Forward primer sequence (5'-3')	Reverse primer sequence (5'-3')
GSDME	TGCAACTTCTAAGTCTGGTGACC	AGTCTGACTCCACAACCACTG
IL-1β	GAAATGCCACCTTTTGACAGTG	TGGATGCTCTCATCAGGACAG
TNF	CTGAACTTCGGGGTGATCGG	GGCTTGTCACTCGAATTTTGAGA
MCP-1	TAAAAACCTGGATCGGAACCAAA	GCATTAGCTTCAGATTTACGGGT
IL-6	CTGCAAGAGACTTCCATCCAG	AGTGGTATAGACAGGTCTGTTGG

Supplementary Table 3. Primers for chip-PCR

Site	Forward primer sequence (5’-3’)	Reverse primer sequence (5’-3’)
Site1	TAGCATAAGCAGGGAGAT	CTGTAATCAAGGGACTGG
Site2	AACAGTAAATACCCAGAG	CACCCTCACTACAGATAA
Site3	CTGCTGCTGTCTCCCTCT	TTTATTGCCCTGGAACCT
Site4	CCAGACTGAAGGGAGAAG	GGTTGGTCGTCGTAGAAA
