# Supplementary material for: GSDME-mediated pyroptosis promotes the progression and associated inflammation of atherosclerosis
Source: Nat Commun. 2023 Feb 18;14:929. doi: 10.1038/s41467-023-36614-w (PMC9938904; doi:10.1038/s41467-023-36614-w)
Supplement: Supplementary file 3 — Reporting Summary [file 41467_2023_36614_MOESM3_ESM.pdf]

nature portfolio

Corresponding author(s): Zuyi Yuan; Yue Wu

Last updated by author(s): Dec 27, 2022

Reporting Summary

Nature Portfolio wishes to improve the reproducibility of the work that we publish. This form provides structure for consistency and transparency in reporting. For further information on Nature Portfolio policies, see our Editorial Policies and the Editorial Policy Checklist.

Statistics

For all statistical analyses, confirm that the following items are present in the figure legend, table legend, main text, or Methods section.

n/a	Confirmed	
☐	☒	The exact sample size (*n*) for each experimental group/condition, given as a discrete number and unit of measurement
☐	☒	A statement on whether measurements were taken from distinct samples or whether the same sample was measured repeatedly
☐	☒	The statistical test(s) used AND whether they are one- or two-sided<br>*Only common tests should be described solely by name; describe more complex techniques in the Methods section.*
☒	☐	A description of all covariates tested
☐	☒	A description of any assumptions or corrections, such as tests of normality and adjustment for multiple comparisons
☐	☒	A full description of the statistical parameters including central tendency (e.g. means) or other basic estimates (e.g. regression coefficient) AND variation (e.g. standard deviation) or associated estimates of uncertainty (e.g. confidence intervals)
☐	☒	For null hypothesis testing, the test statistic (e.g. *F*, *t*, *r*) with confidence intervals, effect sizes, degrees of freedom and *P* value noted<br>*Give P values as exact values whenever suitable.*
☒	☐	For Bayesian analysis, information on the choice of priors and Markov chain Monte Carlo settings
☒	☐	For hierarchical and complex designs, identification of the appropriate level for tests and full reporting of outcomes
☒	☐	Estimates of effect sizes (e.g. Cohen's *d*, Pearson's *r*), indicating how they were calculated

Our web collection on statistics for biologists contains articles on many of the points above.

Software and code

Policy information about availability of computer code

Data collection	no specialized software was used for data acquisition. illumina HiSeq2500 instrument for RNA sequencing; Illumina novaseq 6000 for single-cell RNA sequencing; bcl2fastq (version 2.20) for converting BCL files to FASTQ format; sequencing data were processed using the CeleScope1.1.7 pipeline; Olympus microscope(BX51-FL-CCD)for image acquisition; automatic biochemical analyzer, type 7600 (Hitachi, Tokyo, Japan) for mouse plasma lipid profile;
Data analysis	All software used in this study are cited either in the main text. Here is the list of software used in this study. Harmony v0.1, fastp (version 2.5.3a), featureCounts (version 2.0.1), Seurat package (version 3.1.2), STAR (version 2.6.1a), samtools (version1.9) , Monocle (version 2.14.0) ,'gseapy' python package (version 0.9.15), clusterProfiler (version 3.16.1), JASPAR database (http://jaspar.genereg.net/), Graphpad Prism software (version 8.0), IBM SPSS Statistics 23 software, Quantity one software (version 4.6.2), image J software (version 2.10), Image-Pro Plus 6.0 software (Media Cybernetics, USA).

For manuscripts utilizing custom algorithms or software that are central to the research but not yet described in published literature, software must be made available to editors and reviewers. We strongly encourage code deposition in a community repository (e.g. GitHub). See the Nature Portfolio guidelines for submitting code & software for further information.

Data

Policy information about availability of data

All manuscripts must include a data availability statement. This statement should provide the following information, where applicable:

- Accession codes, unique identifiers, or web links for publicly available datasets
- A description of any restrictions on data availability
- For clinical datasets or third party data, please ensure that the statement adheres to our policy

The raw data of single-cell RNA-seq generated in this study have been deposited in Sequence Read Archive (SRA) database under accession code PRJNA802316 [https://www.ncbi.nlm.nih.gov/bioproject/PRJNA802316]. The raw bulk RNA sequencing data generated in this study have been deposited in Sequence Read Archive (SRA) data base under accession code PRJNA802807 [https://www.ncbi.nlm.nih.gov/bioproject/PRJNA802807]; The publicly data used in this study are available in the GEO database under accession code GSE 43292 [https://www.ncbi.nlm.nih.gov/geo/query/acc.cgi?acc=GSE43292]. The remaining data generated in this study are provided in the Supplementary Information or Source Data file. Source data are provided with this paper.

Human research participants

Policy information about studies involving human research participants and Sex and Gender in Research.

Reporting on sex and gender	The human research participant is not a sex/gender related research and we collected the atherosclerotic plaques were obtained from 8 male and 2 female patients who underwent a carotid endarterectomy (CEA) procedure at The First Affiliated Hospital of Xi'an Jiao tong University after the written informed consent was collected from patients or their relative
Population characteristics	All patients were diagnosed with transient ischemic attack (TIA) or cerebral infarction before surgery accompanied by clinical symptoms such as dizziness or cognization dysfunction. They underwent a carotid endarterectomy (CEA) procedure at The First Affiliated Hospital of Xi'an Jiao tong University. The average age is 60 years. 3 patients had diabetes.5 patients had hypertension. 6 patients had a smoking history , and all the patients had a previous stroke. 4 patients underwent right carotid endarterectomy, and 6 patients underwent left carotid endarterectomy. The detailed medical information of patients with carotid artery stenosis is shown in Supplementary Table 1. No compensation was provided to the participants in this study.
Recruitment	The carotid atherosclerotic plaques were obtained from a carotid endarterectomy (CEA) procedure The written informed consent was collected from patients or their relatives. The plaques were divided into 2 parts, one was fixed in 4% paraformaldehyde and then embedded in optimal cutting temperature compound (SAKURA O.C.T. Compound) and cut to 8-μm sections with a Leica cryostat, the other were quick frozen by liquid nitrogen for protein analysis. we randomly selected these patients, there was no subjective intervention.
Ethics oversight	The study was approved by Research Ethics Committees of The First Affiliated Hospital of Xi'an Jiaotong University (Xi'an, Shaanxi, China)

Note that full information on the approval of the study protocol must also be provided in the manuscript.

Field-specific reporting

Please select the one below that is the best fit for your research. If you are not sure, read the appropriate sections before making your selection.

[x] Life sciences [ ] Behavioural & social sciences [ ] Ecological, evolutionary & environmental sciences

For a reference copy of the document with all sections, see nature.com/documents/nr-reporting-summary-flat.pdf

Life sciences study design

All studies must disclose on these points even when the disclosure is negative.

Sample size	We did not use statistical method to predetermine sample size. The sample size was based on the previous literature that a minimum of N = 3 biological replicates with sufficient reproducibility in cell experiments and a minimum of N = 5 biological replicates with sufficient reproducibility in animal experiments (PMID: 31902237; PMID: 30405100).
Data exclusions	No data were excluded from the analysis.
Replication	All the experiments have been at least replicated for three independent times. All the results have been validated in the independent repeated experiments.
Randomization	The samples for in vivo and in vitro were randomly allocated into different experimental groups.
Blinding	The investigators were blinded to group allocation during experiment, data collection and analysis.

Reporting for specific materials, systems and methods

We require information from authors about some types of materials, experimental systems and methods used in many studies. Here, indicate whether each material, system or method listed is relevant to your study. If you are not sure if a list item applies to your research, read the appropriate section before selecting a response.

Materials & experimental systems

n/a	Involved in the study
☐	☒ Antibodies
☐	☒ Eukaryotic cell lines
☒	☐ Palaeontology and archaeology
☐	☒ Animals and other organisms
☒	☐ Clinical data
☒	☐ Dual use research of concern

Methods

n/a	Involved in the study
☒	☐ ChIP-seq
☒	☐ Flow cytometry
☒	☐ MRI-based neuroimaging

Antibodies

Antibodies used

The following antibodies were used for immunofluorescence and immunohistochemistry staining:
anti-GSDME (Abcam, catalog number: ab230482, 1:100, Rabbit polyclonal),
anti-caspase-3 (CST, catalog number: 9662, 1:1000, Rabbit polyclonal),
anti-IL-1β (Proteintech, catalog number: 66737-1-Ig, 1:200, Clone numbers: 2A1B4),
anti-CD68 (Proteintech, catalog number: 66231-2-Ig, 1:2000, Clone numbers: 3A9A7)
anti-Anti-Monocyte + Macrophage (Abcam, catalog number: ab33451, 1:1000, Clone numbers: MOMA-2),
α-SMA (CST, catalog number: 19245, 1:200, Clone numbers: D4K9N),
anti-CD16 (Abcam, catalog number 183354, 1:100, Clone numbers: SP175),
anti-CD163 (Abcam, catalog number 182422, 1:200, Clone numbers: EPR19518),
Goat Anti-Mouse IgG H&L Cy5 preadsorbed (Abcam, catalog number: ab6563, 1:1000)
Alexa Fluor 488-conjugated goat anti-rabbit (Invitrogen, A-11008, 1:200)
The following antibodies were used for WB:
anti-GSDME (Abcam, catalog number: ab215191, 1:1000, Clone numbers: EPR19859),
anti -caspase-3 (CST, catalog number: 9662, ab230482, 1:1000, Rabbit polyclonal),
anti -α-tubulin (Affinity Biosciences, catalog number: AF7010, Rabbit polyclonal),
anti -STAT3 (CST, catalog number: 12640, 1:1000, Clone numbers: D3Z2G),
anti-phospho-STAT3 (CST, catalog number:9145, 1:1000, Clone numbers: D3A7),
anti -IL-1β (CST, catalog number: 12242, 1:1000, Clone numbers: 3A6)
normal Rabbit IgG (CST, catalog number:2729, 5 μg for a single immunoprecipitation assay),
anti-FLAG (CST, catalog number: 14793, 1:1000, Clone numbers: D6W5B)
anti-GAPDH (Affinity Biosciences, catalog number: AF7021, Rabbit polyclonal),
anti-NLRP3 (Abcam, catalog number: ab270449, 1:1000, Clone numbers: EPR23073-96)
anti-caspase-8 (CST, catalog number:8592 1:1000, Clone numbers: D5B2)
anti-rabbit IgG, HRP-linked Antibody (CST, catalog number:7074, 1:2000)

Validation

All commercial antibody validations are available on manufacturers' websites. Specificity of most of antibodies was validated using wild-type and knockout cells or validated by the manufacturer for reactivity in human tissue with immunofluorescence and/or western blotting.
1) https://www.abcam.cn/dfna5gsdme-antibody-ab230482.html
2) https://www.cellsignal.cn/products/primary-antibodies/caspase-3-antibody/9662?site-search type=Products&N=4294956287&Ntt=9662&fromPage=plp
3) https://www.ptgcn.com/products/IL1-beta-Antibody-66737-1-Ig.htm
4) https://www.ptgcn.com/products/CD68-Antibody-66231-2-Ig.htm
5) https://www.abcam.com/monocyte--macrophage-antibody-moma-2-ab33451.html
6) https://www.cellsignal.cn/products/primary-antibodies/a-smooth-muscle-actin-d4k9n-xp-rabbit-mab/19245?site-search-type=Products&N=4294956287&Ntt=19245&fromPage=plp&_requestid=5706229
7) https://www.abcam.cn/cd16-antibody-sp175-ab183354.html
8) https://www.abcam.cn/cd163-antibody-epr19518-ab182422.html
9) https://www.abcam.cn/dfna5gsdme-antibody-epr19859-n-terminal-ab215191.html
10) https://www.affbiotech.com/goods-6285-AF7010-Tubulin_alpha_Antibody.html
11) https://www.cellsignal.cn/products/primary-antibodies/stat3-d3z2g-rabbit-mab/12640?site-search-type=Products&N=4294956287&Ntt=12640&fromPage=plp&_requestid=574260
12) https://www.cellsignal.cn/products/primary-antibodies/il-1b-3a6-mouse-mab/12242?site-search-type=Products&N=4294956287&Ntt=12242&fromPage=plp&_requestid=5172848
13) https://www.cellsignal.cn/products/primary-antibodies/dykddddk-tag-d6w5b-rabbit-mab-binds-to-same-epitope-as-sigma-s-anti-flag-m2-antibody/14793?site-search-type=Products&N=4294956287&Ntt=14793&fromPage=plp&_requestid=5701254

14) https://www.affbiotech.com/goods-6289-AF7021-GAPDH_Antibody.html
15) https://www.abcam.cn/nlrp3-antibody-epr23073-96-ab270449.html
16) https://www.cellsignal.cn/products/primary-antibodies/cleaved-caspase-8-asp387-d5b2-xp-rabbit-mab-mouse-specific/8592?site-search-type=Products&N=4294956287&Ntt=8592&fromPage=plp&_requestid=5720206

Eukaryotic cell lines

Policy information about cell lines and Sex and Gender in Research

Cell line source(s)	Mouse peritoneal macrophages and mouse bone marrow–derived macrophages were isolated from mice peritoneal cavity and femurs and tibias; 293T cells were purchased from National Infrastructure of Cell Line Resource (Shanghai, China).
Authentication	No authentication has been used.
Mycoplasma contamination	The cells were tested negative for mycoplasma contamination.
Commonly misidentified lines (See ICLAC register)	No commonly misidentified lines were used in the study.

Animals and other research organisms

Policy information about studies involving animals; ARRIVE guidelines recommended for reporting animal research, and Sex and Gender in Research

Laboratory animals	Eight-week-old, male ApoE-/- (C57BL/6 background) and ApoE-/- GSDME-/-mice were used in the study.GSDME-/- mice used in the study were generated by co-microinjection of in vitro-translated Cas9 mRNA and gRNA into the C57BL/6J mice. All mice were housed and bred at the Experiment Animal Center of Xi'an Jiaotong University under pathogen-free conditions.
Wild animals	The study did not involve wild animals.
Reporting on sex	ApoE-/- , GSDME-/-, ApoE-/- GSDME-/- and wild type mice used in our study were all males. We used male mice to constructed atherosclerosis model. We used only male mice in this study given that higher estrogen level in female mice is known to affect macrophage function and thus may complicate our data interpretation. The methods used for our assigning sex is that according to the mice genitals' characteristics at 6 weeks after they born.
Field-collected samples	The study did not involve samples collected from the field.
Ethics oversight	The animal protocol was reviewed and approved by Institutional Ethics Committee for Animal Experiments of Xi'an Jiaotong University.

Note that full information on the approval of the study protocol must also be provided in the manuscript.
